# REVISITING THE TEACHING NURSING HOME: QUALITATIVE ANALYSIS OF NURSING STUDENTS’ CLINICAL EXPERIENCE

**DOI:** 10.1093/geroni/igac059.3070

**Published:** 2022-12-20

**Authors:** Brianna Morgan, Kierra Foley, Nancy Hodgson, Ann Kolanowski, Kim Strauch, Nancy Zionts, Chelsea Dickson, Howard Degenholtz

**Affiliations:** University of Pennsylvania, Philadelphia, Pennsylvania, United States; Penn Nursing, Philadelphia, Pennsylvania, United States; University of Pennsylvania, Philadelphia, Pennsylvania, United States; Penn State University, State College, Pennsylvania, United States; Penn School of Nursing, Philadelphia, Pennsylvania, United States; Jewish Healthcare Foundation, Pittsburgh, Pennsylvania, United States; University of Pittsburgh School of Public Health, Pittsburgh, Pennsylvania, United States; University of Pittsburgh, Pittsburgh, Pennsylvania, United States

## Abstract

Revisiting the Teaching Nursing Home is a two-year pilot project to address the long-term care workforce shortage by introducing nursing students to geriatric nursing while also improving quality of care within nursing homes. The initiative has multiple components: enhanced clinical rotations for nursing students with partner schools of nursing, implementation of the Institute for Healthcare Improvement Age-Friendly Health System “4M” quality improvement model, and an online learning network. Undergraduate and graduate nursing students at three Schools of Nursing participated in clinical experiences at regional nursing homes. Students completed an “activity feedback” form after each clinical rotation at the nursing home or related activity, such as a session about the 4Ms or quality improvement/assessment. The activity feedback form asked students to share their most important takeaway and suggestions for improvement. Data from 340 feedback forms was coded qualitatively using conventional and directed content analysis using the American Association of Colleges of Nursing (AACN) Essentials for Professional Nursing Education. Multiple coders and audit trials were used to establish rigor. Students’ takeaways encompassed 7 of 8 key concepts in the AACN Essentials; Knowledge for Nursing Practice, Person-Centered Care, and Interprofessional Partnerships were most frequently mentioned. Students provided numerous suggestions for improving their clinical experiences including facilitated learning from instructors and supported engagement with nursing home staff. In conclusion, the program addressed many of the core competencies designated by AACN. One recommendation that flows from these findings is to enhance the role of clinical preceptors in the nursing home setting to facilitate mentored training.

